# Cerebral-Protected Percutaneous Thrombectomy for Near-Occlusive LVAD Outflow Graft Thrombosis

**DOI:** 10.1016/j.jaccas.2025.106069

**Published:** 2026-01-28

**Authors:** Teja S. Chakrala, Toochukwu Tanko, Vikram Raje, Lawrence Charles, Matthew Janko, R. Kyle Thompson, Ronnie Ramadan, Ugochukwu O. Egolum

**Affiliations:** a Department of Cardiology, Northeast Georgia Medical Center, Gainesville, Georgia, USA; b Department of Cardiothoracic Surgery, Northeast Georgia Medical Center, Gainesville, Georgia, USA

**Keywords:** cardiac assist devices, cardiomyopathy, chronic heart failure, complication, thrombus

## Abstract

**Background:**

Late low-flow alarms in left ventricular assist devices (LVADs) most commonly result from volume depletion or right-sided failure, but mechanical outflow graft obstruction is an infrequent yet life-threatening cause.

**Case Summary:**

A 70-year-old man with a HeartMate 3 LVAD presented with persistent low-flow (0 L/min) alarms after defecation but felt entirely well. Laboratory testing showed no overt hemolysis, and transthoracic echocardiography excluded right-sided failure. Computed tomographic angiography revealed >90% thrombotic narrowing of the distal outflow graft. The patient underwent percutaneous cerebral-protected thrombectomy with a Penumbra aspiration catheter followed by serial balloon dilatations (8-18 mm) achieving complete graft patency and immediate restoration of device flow to 4 L/min.

**Discussion:**

Outflow graft thrombosis occurs in approximately 2% to 5% of contemporary LVAD recipients and carries high morbidity if untreated. Few reports describe successful catheter-directed thrombectomy; this case highlights prompt multimodality imaging, a heart-team approach, and the utility of cerebral protection to mitigate embolic risk.

**Take-Home Messages:**

Persistent low-flow alarms mandate systematic evaluation. Early imaging-guided intervention can safely rescue outflow graft thrombosis and avoid emergent pump exchange.

## Visual Summary

**Figure:**
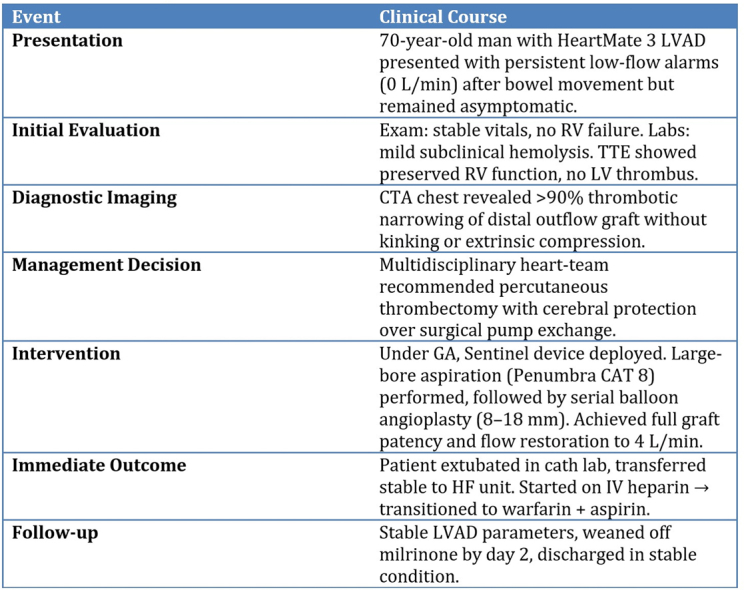

**Visual Summary:**
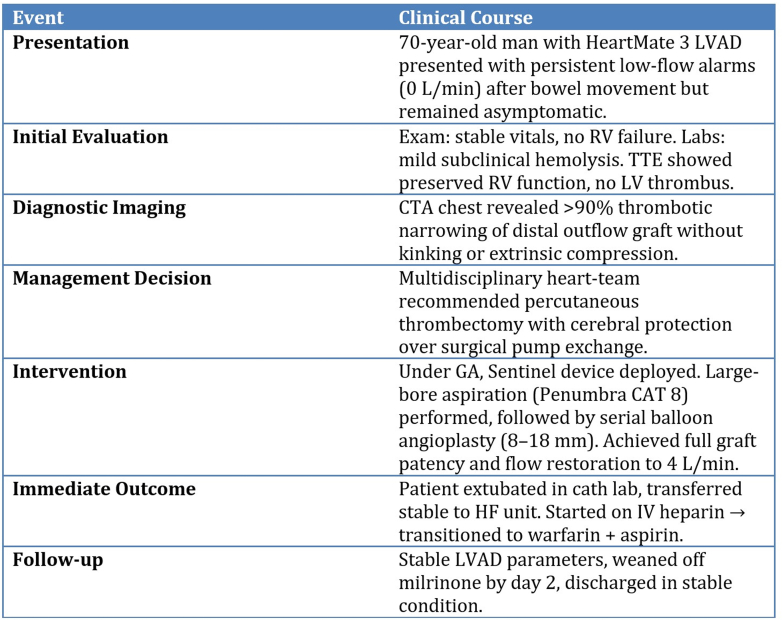
Timeline of Case CTA = computed tomography angiography; GA = general anesthesia; HF = heart failure; IV = intravenous; LVAD = left ventricular assist device; RV = right ventricle; TTE = transthoracic echocardiography.

## History of Presentation

A 70-year-old man with a HeartMate 3 (HM3) left ventricular assist device (LVAD) implanted for ischemic cardiomyopathy noticed repetitive low-flow alarms beginning 4 hours after a routine bowel movement. Displayed flow had fallen from his baseline of 4.5 L/min to 0 L/min. He remained entirely asymptomatic, denying chest pain, dyspnea, light-headedness, hematuria, or melena. He reported generalized fatigue since initiation of levetiracetam for new-onset seizures several months ago and a remote syncopal episode 2 weeks earlier when he bruised the driveline exit site.

**Take-Home Messages**

• Persistent left ventricular assist device low-flow alarms warrant systematic assessment that includes echocardiography and computed tomography angiography to identify mechanical obstruction.
• Percutaneous thrombectomy with cerebral protection can promptly restore graft patency, normalize device flow, and obviate high-risk reoperation.

On examination, he was afebrile and had a heart rate of 62 beats/min, a blood pressure of 98/62 mm Hg, and an oxygen saturation of 98% on ambient air. A continuous LVAD hum was audible. Jugular venous pressure was not elevated, lungs were clear, and there was no peripheral edema or neurologic deficit.

## Past Medical History

The patient had a past medical history of ischemic cardiomyopathy, coronary artery bypass grafting (2015), HM3 LVAD (January 27, 2020) complicated by driveline infection (March 2025), chronic kidney disease stage IIIa, paroxysmal atrial fibrillation status post pulmonary vein isolation; nonsustained ventricular tachycardia status post dual-chamber implantable cardioverter defibrillator (2019), carotid artery disease status post carotid endarterectomy, chronic combined systolic and diastolic heart failure, hyperlipidemia, and seizure disorder.

Home medications included bisoprolol 7.5 mg twice a day, losartan 75 mg daily, eplerenone 25 mg daily, and warfarin 2 mg daily (goal international normalized ratio [INR]: 2.0-3.0).

## Differential Diagnosis

Key causes of LVAD low-flow alarms considered were hypovolemia or acute bleeding, right ventricular (RV) failure or tamponade, inflow cannula obstruction due to thrombus or malposition, outflow graft obstruction (twist, thrombus, and kinking), and pump thrombosis or controller malfunction. In this case, extrinsic compression was excluded on computed tomography angiography (CTA), which demonstrated intraluminal thrombus without external mass effect or graft kinking.

## Investigations

Admission chemistry showed creatinine 1.44 mg/dL, blood urea nitrogen 19 mg/dL, hemoglobin 14.5 g/dL, lactate dehydrogenase 272 U/L, plasma-free hemoglobin 110 mg/L (mildly elevated), and normal haptoglobin (70 mg/dL), suggesting subclinical—not overt—hemolysis. INR was 2.52. N-terminal pro–B-type natriuretic peptide had improved from 4,706 pg/mL (prior admission) to 2,711 pg/mL.

Initial LVAD parameters were speed 5,350 rpm, power 3.3 W, pulsatility index 1.8, and flow 0.0 L/min despite adequate battery voltage. Controller exchange transiently increased flow to 0.2 L/min, arguing against pump thrombosis. Transthoracic echocardiography demonstrated midline interventricular septum, no left ventricular thrombus, and aortic valve opening with every beat. In addition, RV size/function appeared preserved, and there was no pulmonary hypertension present.

**Figure 1:**
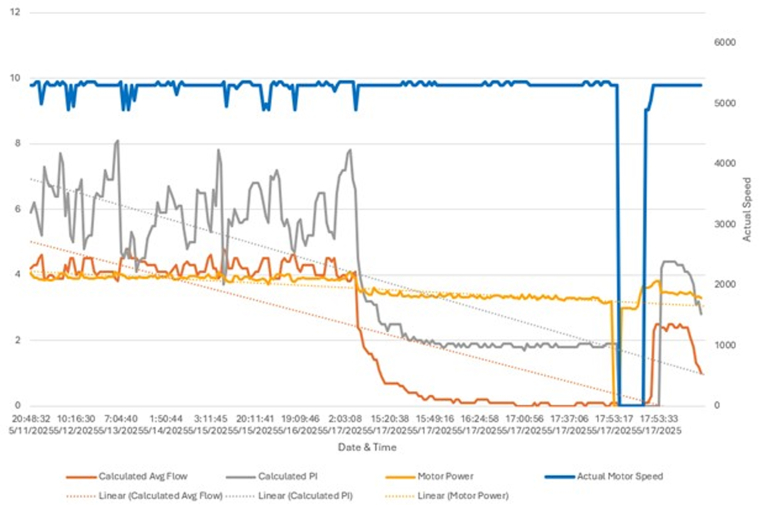
LVAD HM3 Event Log File LVAD HM3 event log chart demonstrating downward trends of PI and power consistent with interference and obstruction to outflow. HM3 = HeartMate 3; LVAD = left ventricular assist device; PI = pulsatility index.

**Figure 2:**
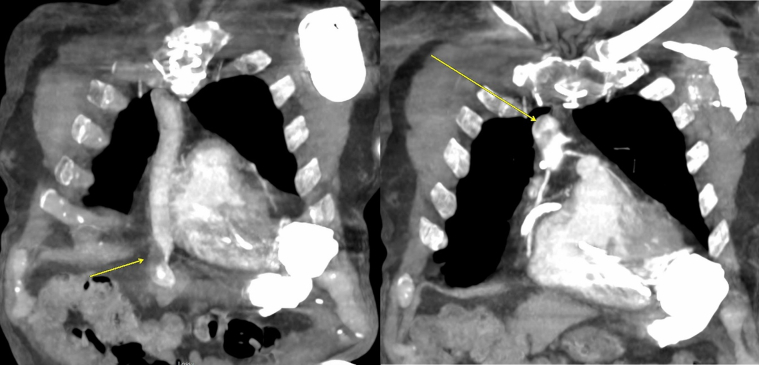
CTA Chest Images CTA chest demonstrating near-occlusive thrombus in LVAD outflow graft. CTA = computed tomography angiography; LVAD = left ventricular assist device.

Given persistent alarms and a downward power trend noted on log files (Figure 1), CTA of the chest was obtained, revealing a near-occlusive crescentic thrombus in the distal outflow graft with >90% luminal narrowing and no kinking (Figure 2, Video 1, Video 2, Video 3).

## Management

### Medical stabilization

The patient received 500 mL of isotonic saline without improvement in flow. Intravenous milrinone (0.25 μg/kg/min) was initiated to augment cardiac output, and unfractionated heparin was started (target activated partial thromboplastin time 60-80 seconds), while warfarin was held.

### Heart-team decision

After multidisciplinary review, percutaneous thrombectomy with adjunctive balloon angioplasty was favored over surgical pump exchange because of the focal nature of obstruction, absence of device thrombosis, and acceptable surgical risk profile. To mitigate cerebral embolization, transradial Sentinel cerebral protection was planned.

### Interventional procedure

**Figure 3:**
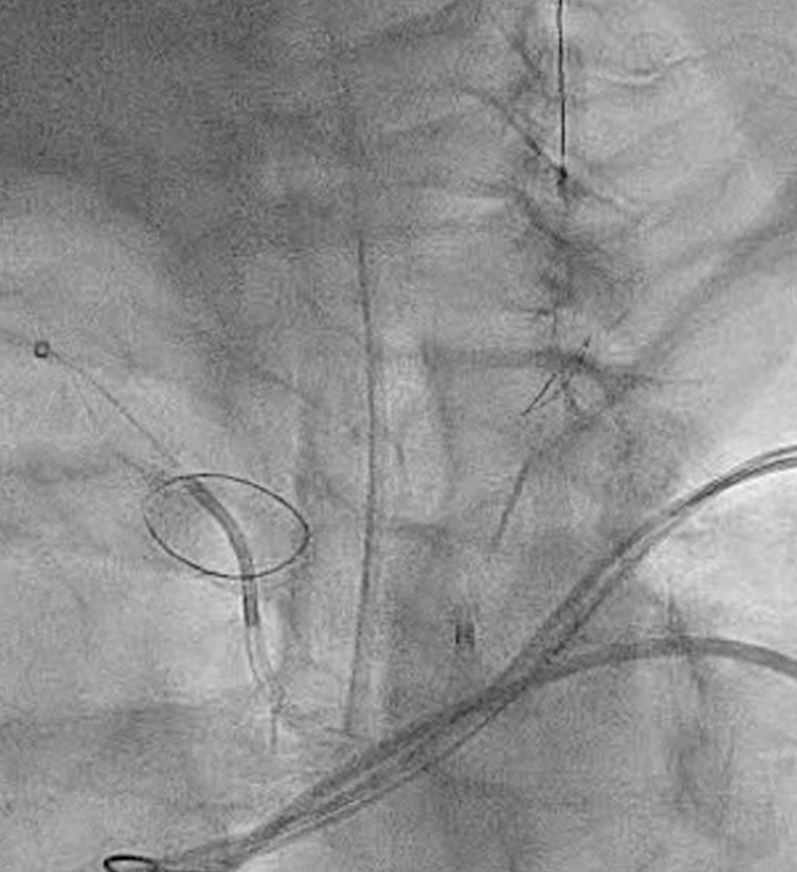
Cerebral Protection Device Deployment on Fluoroscopy Sentinel cerebral embolic protection device deployment before thrombectomy.

**Figure 4:**
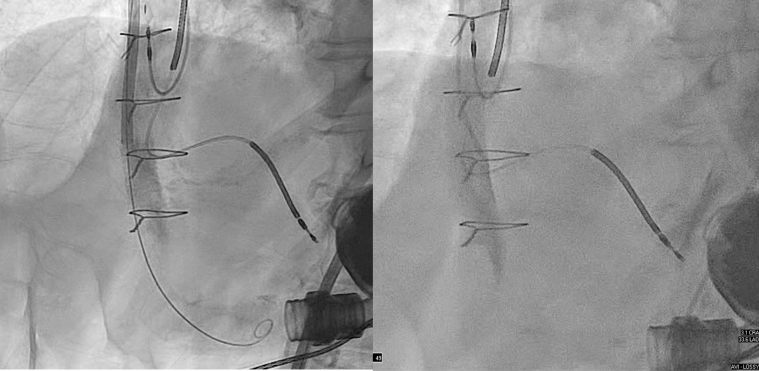
Preprocedure Angiography of LVAD Outflow Graft Angiography of LVAD outflow graft demonstrating occlusive thrombus. LVAD = left ventricular assist device.

**Figure 5:**
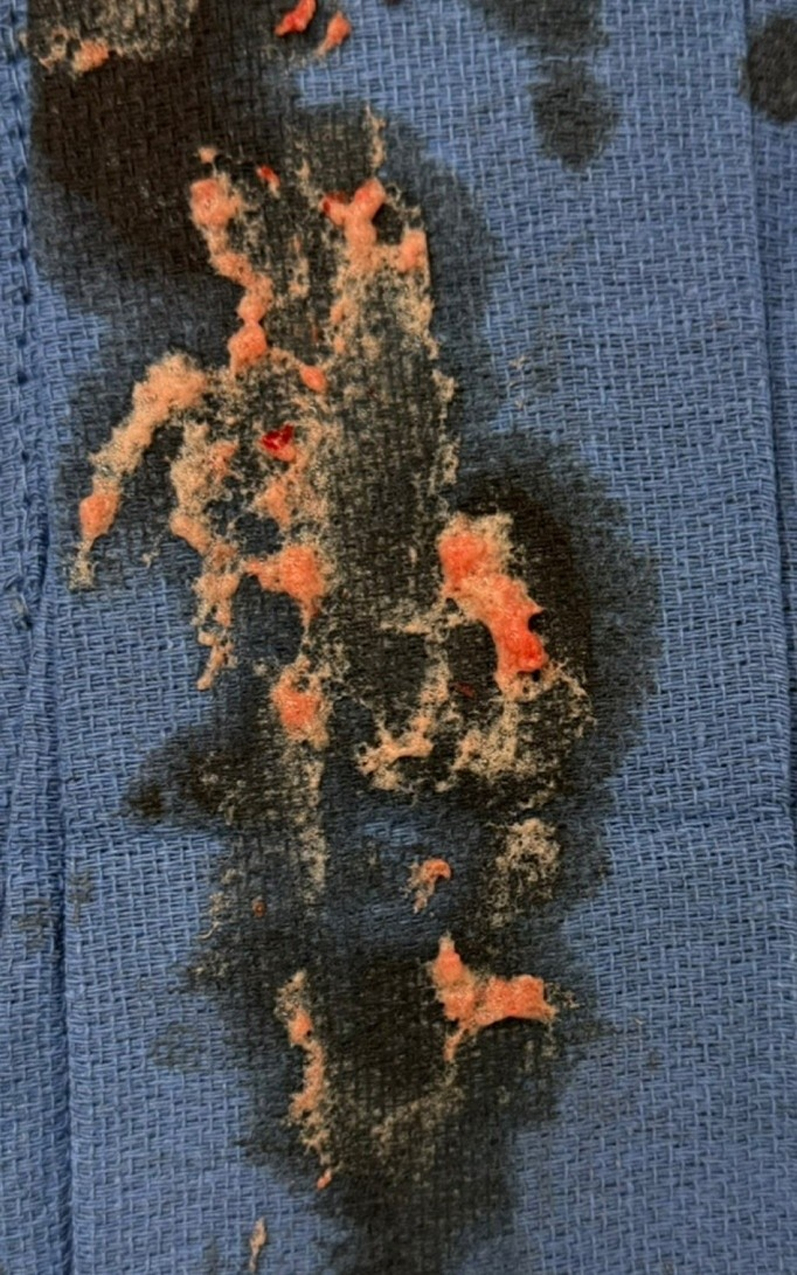
Thrombus Material Thrombus material aspirated from left ventricular assist device outflow graft.

**Figure 6:**
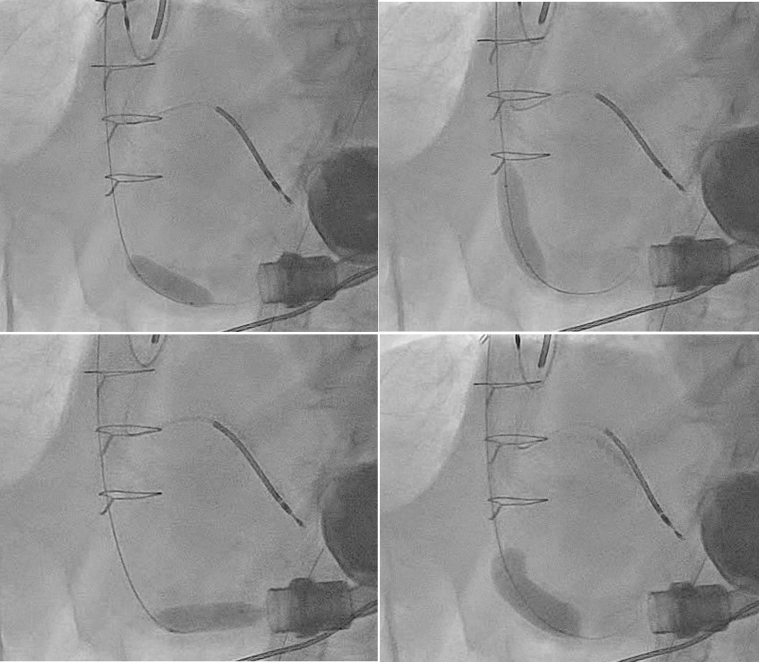
Balloon Angioplasty of LVAD Outflow Graft Serial balloon dilation of LVAD outflow graft. LVAD = left ventricular assist device.

**Figure 7:**
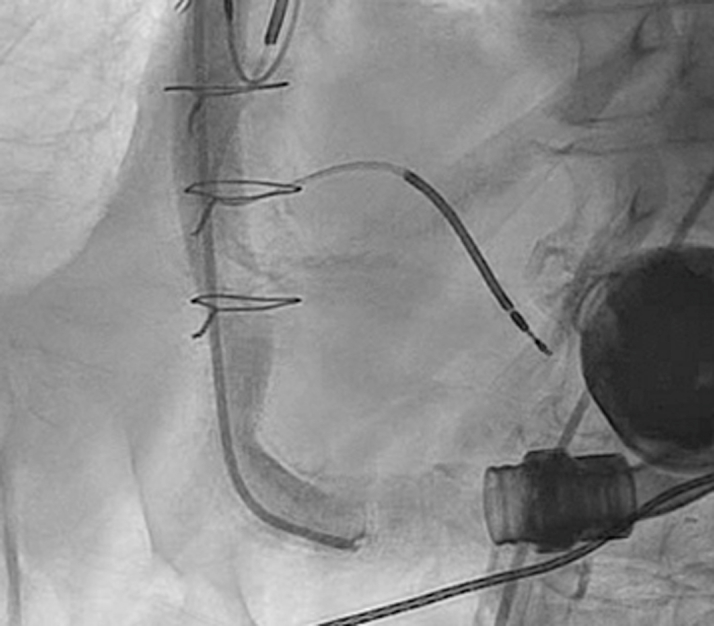
Postprocedure Angiography of Left Ventricular Assist Device Outflow Graft Postballoon dilation angiography demonstrating restored flow through outflow graft.

Under general anesthesia, right radial and bilateral femoral arterial access was obtained. After Sentinel deployment in the brachiocephalic and left common carotid arteries (Figure 3), the LVAD outflow graft was selectively cannulated via a 16-F Gore DrySeal sheath. Angiography of the LVAD outflow graft was performed, demonstrating occlusive thrombus (Figure 4, Videos 4 and 5). Aspiration thrombectomy was performed with a Penumbra CAT 8 device; multiple passes extracted white fibrin-rich material (Figure 5) with modest blood loss. Residual luminal compromise was treated with sequential 8-, 14-, and 18-mm noncompliant balloon inflations (Figure 6, Video 6, Video 7, Video 8, Video 9), achieving a widely patent channel on completion angiography (Figure 7, Video 10). LVAD speed was titrated to 5,300 rpm with an immediate rise in flow to 4 L/min, power 3.8 W, and pulsatility index 5.5. Access sites were closed with Perclose sutures without complication.

### Outcome and follow-up

The patient was extubated in the catheterization laboratory and transferred to the heart failure unit in a hemodynamically stable condition. Heparin was transitioned to warfarin (target INR: 2.5-3.0) plus aspirin 81 mg. Milrinone was weaned on day 2; flow and power remained stable.

### Conclusions

Outflow graft thrombosis should be suspected when low-flow alarms persist despite normal pump speed, stable hemodynamics, and absence of RV failure. Rapid multimodal imaging differentiates mechanical from physiological causes and helps guide therapy. Catheter-based thrombectomy coupled with high-pressure balloon angioplasty is an effective, minimally invasive alternative to surgical graft revision or pump exchange, particularly when combined with cerebral protection to lower stroke risk. Early heart-team collaboration is essential to expedite diagnosis and definitive intervention.

## Discussion

Near occlusive HM3 outflow graft thrombosis is an infrequent but increasingly recognized cause of late low flow alarms as support durations extend. Abbott's 2024 field safety notice, based on MOMENTUM 3 surveillance, estimates the cumulative incidence of biodebris-related obstruction at 0.24% by 2 years and 2.06% by 5 years, though single-center series report overall post-pump obstruction rates nearer 2% to 5%.^1^ Pathogenesis is multifactorial; hydrodynamic modeling and pathologic data show progressive deposition of proteinaceous biodebris between the graft and its bend relief, creating a relative stenosis that promotes local flow stagnation; superimposed platelet rich thrombus can then precipitate rapid near occlusion.^2^ In a contemporary cohort, thrombus accounted for 34.6% of outflow obstructions, concentric stenosis for a similar proportion, and kinking for 11.5%.^3^

Because clinical manifestations overlap those of intrapump thrombosis, a structured diagnostic algorithm is essential. Ramp transthoracic echocardiography demonstrating reduced unloading and a falling pump power trend raises suspicion, but CTA remains the gold standard, distinguishing eccentric, high attenuation thrombus from concentric biodebris and excluding twist or kinking.^2^ Our patient exemplifies the value of early CTA: despite silent systemic perfusion and minimal hemolysis, imaging disclosed >90% distal graft narrowing, directing therapy away from empiric volume resuscitation or thrombolysis.

Historically, redo sternotomy for pump exchange offered definitive management but carries 30-day mortality exceeding 25% in modern series, largely because of adhesiolysis and RV failure.^2^ Less invasive options include systemic thrombolysis, endovascular stenting, and mechanical thrombectomy. Thrombolytics achieve prompt flow restoration but are limited by intracranial hemorrhage rates of 11% to 12% in LVAD recipients.^4^ Covered stent implantation normalizes flows acutely in roughly 86% of HM3 cases, yet the largest patient level meta-analysis (n = 28) documented 12% 30-day mortality and an 8% rate of recurrent stenosis, neurologic events, or surgical revision within 4 months.^5^ Moreover, nearly 29% experienced stent placement failure, especially when residual luminal patency was <3 mm or the lesion involved complex twisting.^6^

Mechanical or aspiration thrombectomy offers a compelling alternative for fresh thrombus by restoring native graft caliber while avoiding a permanent prosthesis. Published experience, however, remains limited to isolated reports—often with extracorporeal membrane oxygenation backup—demonstrating rapid hemodynamic recovery and discharge in hemodynamically compromised patients.^7^ Our case adds several important insights. First, even complete “flat line” flow alarms may be clinically silent, underscoring the need to treat device metrics with the same urgency as patient symptoms. Second, large-bore aspiration (Penumbra CAT 8) combined with sequential high-pressure balloon angioplasty achieved immediate restoration of 4 L/min flow without extracorporeal membrane oxygenation, suggesting that standalone thrombectomy is feasible when obstruction is focal and distal access is unimpeded. Third, transient LVAD speed reduction to 3,000 rpm during aspiration minimized competitive forward flow and facilitated clot capture—an approach borrowed from stent series protocols. Finally, routine deployment of the Sentinel cerebral protection device intercepted visible debris and likely contributed to the absence of periprocedural stroke; a 6-patient case series reported 100% debris capture and no neurologic events using the same strategy.^6^

Selection of the optimal percutaneous technique should therefore be lesion specific. Eccentric thrombus causing abrupt caliber loss, as in our patient, is best treated with aspiration ± angioplasty; residual recoil or concentric biodebris may then be scaffolded with a covered stent in the same session.^8^ Conversely, long segment stenosis from chronic biodebris or external twist typically necessitates primary stenting, accepting the small but tangible risk of late restenosis and dual antiplatelet therapy. Thrombolysis is now largely reserved for patients who are moribund or lack catheter facilities.^9^

Important knowledge gaps persist. No prospective registry tracks thrombectomy durability, optimal catheter size, or anticoagulation strategy after the procedure. Cerebral protection devices add cost and radial access but have yet to be tested in a randomized fashion. Device innovation—flexible, large lumen aspiration catheters with integrated distal filters—may further mitigate embolic risk. Until such data emerge, best practice rests on early multimodality imaging, prompt heart team deliberation, and meticulous neuroprotection for any percutaneous manipulation of thrombus. Although cerebral protection reduces neurologic complications, the possibility of systemic embolization to other vascular territories such as mesenteric or renal arteries persists, highlighting the need for meticulous procedural technique and close postintervention surveillance.

In summary, this case demonstrates that cerebral protected percutaneous thrombectomy, supplemented by balloon angioplasty, can rescue near occlusive HM3 outflow graft thrombosis rapidly and safely, sparing the morbidity of surgery and the limitations of stenting. As LVAD prevalence rises and device longevity extends, integration of thrombectomy into the therapeutic algorithm offers a valuable, minimally invasive option for appropriately selected patients.

## Funding Support and Author Disclosures

The authors have reported that they have no relationships relevant to the contents of this paper to disclose.

## Appendix

Video 1 Computed Tomography Angiography of the Chest Demonstrating Occlusive Thrombus of the Left Ventricular Assist Device Outflow Graft

Video 2 Computed Tomography Angiography of the Chest Demonstrating Occlusive Thrombus of the Left Ventricular Assist Device Outflow Graft

Video 3 Computed Tomography Angiography of the Chest Demonstrating Occlusive Thrombus of the Left Ventricular Assist Device Outflow Graft

Video 4 Preprocedure Angiography Demonstrating Left Ventricular Assist Device Outflow Graft Obstruction

Video 5 Preprocedure Angiography Demonstrating Left Ventricular Assist Device Outflow Graft Obstruction

Video 6 Serial Balloon Dilatation and Angioplasty of Left Ventricular Assist Device Outflow Graft

Video 7 Serial Balloon Dilatation and Angioplasty of Left Ventricular Assist Device Outflow Graft

Video 8 Serial Balloon Dilatation and Angioplasty of Left Ventricular Assist Device Outflow Graft

Video 9 Serial Balloon Dilatation and Angioplasty of Left Ventricular Assist Device Outflow Graft

Video 10 Postprocedure Angiography Demonstrating Restored Left Ventricular Assist Device Outflow Graft Flow
